# Postmortem Brains from Subjects with Diabetes Mellitus Display Reduced GLUT4 Expression and Soma Area in Hippocampal Neurons: Potential Involvement of Inflammation

**DOI:** 10.3390/cells12091250

**Published:** 2023-04-25

**Authors:** Caio Yogi Yonamine, Marisa Passarelli, Claudia Kimie Suemoto, Carlos Augusto Pasqualucci, Wilson Jacob-Filho, Venâncio Avancini Ferreira Alves, Suely Kazue Nagahashi Marie, Maria Lucia Correa-Giannella, Luiz Roberto Britto, Ubiratan Fabres Machado

**Affiliations:** 1 Department of Physiology and Biophysics, Institute of Biomedical Sciences, University of São Paulo, São Paulo 05508-000, Brazil; 2 Novo Nordisk Foundation Center for Basic Metabolic Research, Faculty of Health and Medical Sciences, University of Copenhagen, DK-2200 Copenhagen, Denmark; 3 Laboratório de Lipides (LIM-10) do HCFMUSP, Faculdade de Medicina, Universidade de São Paulo, São Paulo 01246-000, Brazil; 4 Programa de Pos-Graduação em Medicina, Universidade Nove de Julho (UNINOVE), São Paulo 01525-000, Brazil; 5 Divisao de Geriatria, Faculdade de Medicina, Universidade de Sao Paulo, São Paulo 01246-000, Brazil; 6 Departamento de Patologia, Universidade de Sao Paulo, São Paulo 01246-000, Brazil; 7 Laboratório de Investigação Médica em Patologia Hepática, (LIM14) do Hospital das Clínicas (HCFMUSP), Faculdade de Medicina, Universidade de São Paulo, São Paulo 01246-000, Brazil; 8 Departamento de Neurologia, Faculdade de Medicina, Universidade de Sao Paulo, São Paulo 01246-000, Brazil; 9 Laboratorio de Carboidratos e Radioimunoensaio (LIM-18) do Hospital das Clinicas HCFMUSP, Faculdade de Medicina, Universidade de Sao Paulo, São Paulo 01246-000, Brazil

**Keywords:** GLUT4, diabetes, obesity, human hippocampus, SH-SY5Y cell, neurodegeneration, Alzheimer’s disease, inflammation, histone acetylation, HDAC3 inhibition

## Abstract

Diabetes mellitus (DM) is an important risk factor for dementia, which is a common neurodegenerative disorder. DM is known to activate inflammation, oxidative stress, and advanced glycation end products (AGEs) generation, all capable of inducing neuronal dysfunctions, thus participating in the neurodegeneration progress. In that process, disturbed neuronal glucose supply plays a key role, which in hippocampal neurons is controlled by the insulin-sensitive glucose transporter type 4 (GLUT4). We investigated the expression of GLUT4, nuclear factor NF-kappa B subunit p65 [NFKB (p65)], carboxymethyllysine and synapsin1 (immunohistochemistry), and soma area in human postmortem hippocampal samples from control, obese, and obese+DM subjects (41 subjects). Moreover, in human SH-SY5Y neurons, tumor necrosis factor (TNF) and glycated albumin (GA) effects were investigated in GLUT4, synapsin-1 (SYN1), tyrosine hydroxylase (TH), synaptophysin (SYP) proteins, and respective genes; NFKB binding activity in the *SLC2A4* promoter; effects of increased histone acetylation grade by histone deacetylase 3 (HDAC3) inhibition. Hippocampal neurons (CA4 area) of obese+DM subjects displayed reduced GLUT4 expression and neuronal soma area, associated with increased expression of NFKB (p65). Challenges with TNF and GA decreased the *SLC2A4*/GLUT4 expression in SH-SY5Y neurons. TNF decreased *SYN1*, *TH,* and *SYP* mRNAs and respective proteins, and increased NFKB binding activity in the *SLC2A4* promoter. Inhibition of HDAC3 increased the *SLC2A4* expression and the total neuronal content of CRE-binding proteins (CREB/ICER), and also counterbalanced the repressor effect of TNF upon these parameters. This study revealed reduced postmortem human hippocampal GLUT4 content and neuronal soma area accompanied by increased proinflammatory activity in the brains of DM subjects. In isolated human neurons, inflammatory activation by TNF reduced not only the *SLC2A4*/GLUT4 expression but also the expression of some genes related to neuronal function (*SYN1*, *TH*, *SYP*). These effects may be related to epigenetic regulations (H3Kac and H4Kac status) since they can be counterbalanced by inhibiting HDAC3. These results uncover the improvement in GLUT4 expression and/or the inhibition of HDAC3 as promising therapeutic targets to fight DM-related neurodegeneration.

## 1. Introduction

Dementia is a progressive neurodegenerative disorder, the main symptoms of which include loss of memory, thinking, and behavior [1]. Among various subtypes of dementia, Alzheimer’s disease (AD) is the most common. The amyloid hypothesis is a classical theory to explain the mechanisms involved in AD neurodegeneration; however, several efforts aiming to reduce amyloid beta protein accumulation have failed to promote some significant clinical benefits in patients with AD [2,3]. Since the Rotterdam Study, which demonstrated that diabetes mellitus (DM) is an important risk factor for the development and progression of dementia [4], the theory that DM plays a significant role in the pathogenesis of AD has been strengthened by epidemiological, neuropathological, and clinical evidence [5,6,7]. Moreover, the Hisayama Study revealed that hippocampal atrophy is associated with long DM duration and elevated glycemia two hours after glucose overload [8,9], and several meta-analyses have strengthened the higher risk of subjects with DM to develop AD [10,11]. Furthermore, evidence shows that DM promotes amygdala and hippocampus atrophy in non-demented elderly subjects [12] and induces a mild cognitive impairment condition twice as fast as in healthy subjects [13]. Finally, a systematic review of proteomic studies has pointed out several biomarkers common to AD and DM, indicating that these diseases match some similar molecular bases [11].

In addition to the participation of DM in the pathogenesis of AD, obesity has also been suggested as a risk factor for AD [14]. Excessive body fat was found to be directly associated with cognitive decline [15] and overweight and obesity in young adults were associated with reduced gray matter density [16]. In obese subjects, insulin resistance (IR) predisposes to the development of type 2 DM (T2D); furthermore, IR can extend to the central nervous system (CNS), and reduced cerebral glucose utilization has been associated with increased AD risk [17]. Indeed, evidence has repeatedly revealed that central IR plays an important role in the development/progression of AD [18,19]. 

Obesity, IR, and DM are known to be related to inflammation, oxidative stress, mitochondrial damage, and advanced glycation end products generation (AGEs), all these mechanisms are capable of inducing neuronal dysfunctions; thus, they are considered important risk factors for AD [11,18,20]. In this process, it is important to highlight that appropriate neuronal glucose supply is fundamental to guarantee neuronal homeostasis [21]. In vitro studies measuring electrophysiological responses of hippocampal slices exposed to glucose deprivation revealed a reduction in evoked field excitatory post-synaptic potentials, induction, and maintenance of long-term potentiation and NADH response in the cornu ammonis 1 area (CA1) of the hippocampus [22,23].

In the CNS, the neuronal glucose supply is a complex task. In short, glucose firstly leaves the capillary blood through the blood–brain barrier (BBB) by a transendothelial transport mediated by the glucose transporter type 1 (GLUT1) [24,25]. Thus, glucose accesses the neurons through the glucose transporter type 3 (GLUT3) in most brain areas, or through the glucose transporter type 4 (GLUT4) in some specific areas such as the hippocampus [25]. GLUT4 is the typical glucose transporter isoform of adipose and muscle tissues, which can be acutely translocated from the intracellular to the plasma membrane, rapidly increasing the glucose influx [24,25]. In hippocampal neurons, insulin [26] and electrical stimulation [27] also drive the recruitment of GLUT4 to the plasma membrane, increasing the neuronal glucose supply. Concomitantly, these stimuli activate glycolytic enzymes, guaranteeing the neuronal ATP demand [28,29].

Neuroglycopenia is a powerful inducer of brain damage development, and BBB GLUT1 deficiency syndrome is a prototypical brain energy failure [30]. Initially described as a developmental encephalopathy characterized by infantile onset of refractory epilepsy, GLUT1 deficiency syndrome has undergone a dramatic expansion in the range of abnormalities, including mild phenotypes of cognitive impairment [31,32]. On the other hand, peripheral blood glucose lowering also compromises central glucose disposal such as nesidioblastosis and insulinoma, which are associated with hypoglycemia-induced neuroglycopenic symptoms, including acute or permanent impairment in mental status, cognition, and memory and the risk of permanent brain injury [33,34]. Furthermore, in either type 1 DM (T1D) or T2D subjects, treatment with insulin or insulin secretagogues can induce recurrent episodes of hypoglycemia, leading to brain neuronal dysfunction and increasing cognitive impairment and dementia development [35,36].

These data together reinforce the importance of adequate glucose supply to prevent neuronal degeneration, which, in the hippocampus, despite DM-induced hyperglycemia, depends on the adequate expression and function of GLUT4. Indeed, brain insulin resistance or reduced insulin levels contribute to neuronal loss and plasticity development and have been related to impaired cognition, learning, and memory [37,38,39]. Moreover, intrahippocampal inhibition of GLUT4-mediated glucose transport in rats impairs memory acquisition, strengthening GLUT4 as a key regulator of hippocampal memory processing [26].

Systemic inflammation, such as that occurring in DM, can trigger CNS inflammation, an event commonly observed in neurodegenerative processes [40,41]. Tumor necrosis factor protein (TNF) has received special attention in that regard. Although TNF enhances synaptic efficacy at physiological levels [42], at increased levels, it can induce deleterious cellular responses, leading to hippocampal dysfunctions [43,44]. In humans with mild to severe AD, high serum levels of TNF are associated with a twofold increase in the rate of cognitive decline over six months [45]; although, this association has not been observed in other studies [46]. Importantly, the activation of inflammatory pathways in muscle or adipose tissue was reported to inhibit the expression of the *Slc2a4* (solute carrier family 2 member 4) gene, which encodes the GLUT4 protein [47,48,49,50,51].

Finally, in the context of DM/inflammation, increasing generation of advanced glycation end products (AGEs) may be involved in brain damage. AGE interaction with the advanced glycation end product receptor (RAGE) activates pro-inflammatory signals, creating a vicious cycle [52]. Indeed, recent studies have demonstrated that several brain inflammatory conditions are accompanied by increased AGEs activity and RAGE expression in the hippocampus [53,54]. Interestingly, the muscles of rats chronically treated with AGE-albumin exhibit increased proinflammatory activity and reduced *Slc2a4*/GLUT4 content [50].

Although several studies have focused on the relationship between brain injury and IR/inflammation/AGEs, up to the present, no study has clearly associated that relationship with the participation of GLUT4 as an important step for glucose supply to the hippocampal neurons. In muscle and adipose tissues, these conditions are described to be directly involved in the repression of *Slc2a4* gene expression, in an epigenetic and non-epigenetic manner, reducing the cellular GLUT4 protein content and compromising the cellular glucose influx [55], a regulation that might be similarly operating in hippocampal neurons.

Considering accumulated evidence that 1. DM increases the risk for AD development [8,9,10]; 2. normal glucose supply is essential to preserve hippocampal neuron function [22,23]; and 3. GLUT4 is a key player in the hippocampal neuron glucose uptake [56], we hypothesize that impaired GLUT4 expression may be highly involved in the pathogenesis of DM-related neurodegeneration. Thus, the present study investigated whether the GLUT4 protein expression is altered in the postmortem hippocampus of obese and obese/DM human subjects. Moreover, the involvement of TNF and AGEs in the *Slc2a4*/GLUT4 expression, as well as the effect of histone deacetylase 3 (HDAC3) inhibition upon *SLC2A4* expression, was investigated in cultivated SH-SY5Y cells, attempting to reveal that GLUT4-related epigenetic and non-epigenetic mechanisms are potentially involved in the development and/or progression of neurodegeneration.

## 2. Materials and Methods

### 2.1. Subjects

Forty-one subjects from the Biobank for Aging Studies (BAS) of Sao Paulo Medical School were selected. The autopsies were performed at the Sao Paulo Autopsy Service (SPAS). All procedures were approved by the Ethical Board of the School of Medicine (University of Sao Paulo) and an informed consent form was signed by each next-of-kin [57]. The deceased`s relatives, who had at least weekly contact with the deceased for six months until death, were interviewed by an expert team to obtain detailed information about sociodemographic data, history of diseases, lifestyle, and cognitive function status. Up to 24 h after death, samples from the brain hippocampal area were obtained. Samples of the hippocampus were embedded in paraffin and sectioned to generate 5 µm thick slices for molecular and histological analyses. The slices were stored until further analysis described in the next subsection. The samples from the forty-one subjects were divided into three groups according to their body mass index (BMI) and the history of DM: overweight (BMI from 25 to 29.9 kg/m^2^) and obese (BMI from 30 to 34.9 kg/m^2^) with or without DM. The overweight group was considered the control group. 

### 2.2. Immunohistochemistry (IHC) for Quantification of Proteins in Human Hippocampal Area

Paraffin was taken off by means of 30 min xylol incubation for three times at 60 °C, followed by rehydration. The epitope/antigen retrieval was induced by heated acetic acid solution (10%), incubation (10 min), and washing three times (10 min each) with 0.01 M phosphate buffer (PB) solution, following overnight incubation (16 h) with primary antibody solution containing 5% normal donkey serum in 0.3% Triton X-100 in PB diluted 1:250. Then, the slices were washed three times for 10 min with PB, following incubation for 2 h with a biotinylated secondary IgG antibody diluted at 1:200 in PB containing 0.3% Triton X-100 (Jackson Labs, West Grove, PA, USA). Once again, the sections were washed three times (10 min each) with PB, following incubation for 2 h with the avidin–biotin–peroxidase complex (ABC Elite; Vector Labs, Burlingame, CA, USA). Then, the slices were incubated with 3-3-diaminobenzidine tetrahydrochloride-DAB (0.05%) and hydrogen peroxide diluted in PB (0.01%). The slices were mounted on slides and kept overnight at 37 °C. The material was dehydrated and stained with Giemsa (for p65 and synapsin-1 analysis) or hematoxylin (for GLUT4 and carboxymethyllysine analysis), cleared with xylol, and coverslipped with Permount (Fisher, Pittsburgh, PA, USA). Images were taken using Nikon microscope model E1000, Nikon DMX1200 digital camera and Nikon Imaging Software ACT-U (Nikon Instruments Inc., Melville, NY, USA). The integrated density of stained proteins and determination of neuronal density and average neuron size were manually quantified using Image J software (National Institutes of Health, Bethesda, MD, USA version 1.51). Additional details about the IHC and the primary antibodies used are presented in Supplementary Material. The negative control of immunostaining was the absence of a signal after primary antibody removal from the procedure. The protein content results were expressed as arbitrary units, related to mean of the controls, which was set as 1.0. The neuronal density was expressed by a number of neurons/mm^2^, and the average neuronal soma area was expressed by µm^2^.

### 2.3. Cell Culture and Treatments

The SH-SY5Y cells (ATCC) were obtained from the Rio de Janeiro Cell Bank and stained by trypan blue protocol according to manufacturer recommendations and manually counted using a Neubauer chamber (ThermoScientific, Rockford, IL, USA, #15250061). They were seeded at 2 × 10^4^ cells/cm^2^ and cultured in EMEM/F-12 (1:1) medium containing 10% of FBS (fetal bovine serum) and 1% of antibiotics (Penicillin–Streptomycin). After 24 h, the medium was carefully renewed by EMEM/F-12 (1:1) medium containing 3% of FBS, 1% of antibiotics, and 10 µM of retinoic acid (Sigma-Aldrich, St. Louis, MO, USA, #R2625) to induce cell differentiation. After 96 h, the evaluation of differentiation efficacy was performed by measuring the gene expression of synaptophysin (*SYP*), synapsin-1 (*SYN1*), and tyrosine hydroxylase (*TH*) by quantitative polymerase chain reaction (qPCR) and by subjective identification of neurite outgrowth projections as shown in Figure S1. Immediately after cell differentiation, SH-SY5Y cells were subjected to four different experimental design protocols: (1) tumor necrosis factor (TNF) (Sigma-Aldrich, #H8916) at 20 ng/mL for 3 h; (2) glycated albumin (GA) or non-glycated albumin at 0.4 mg/mL for 24 h; (3) RGFP-996 (HDAC3 inhibitor) at 10 µM (Abcam, UK, #ab144819) for 3, 6, 12, and 24 h; or (4) TNF at 20 ng/mL with or without 10 µM RGFP-996 for 6 h. The GA was obtained as previously described [50]. Following the treatments, the cells were subjected to molecular analyses described in the next sub-sections. After all experimental treatments, the reduction in yellow tetrazolium salt 3-(4,5-dimethylthiazol-2-yl)-2,5-diphenyltetrazolium bromide (MTT) was performed to evaluate cellular viability as previously described [49].

### 2.4. Quantitative Polymerase Chain Reaction (qPCR) for Gene Expression Analysis

Total RNA from SH-SY5Y cells was extracted using TRIzol^®^ Reagent according to manufacturer recommendations (Invitrogen, Carlsbad, CA, USA) and the reverse transcriptase reaction was performed using random primers (Invitrogen, Carlsbad, CA, USA), and ImProm-II^®^ Reverse Transcription System (Promega Corporation, Madison, WI, USA). Then, the quantitative PCR amplification was performed using PowerUp^®^ SYBR^®^ Green Master Mix (Applied Biosystems Inc., Foster City, CA, USA), by means of a StepOne Plus Instrument (Applied Biosystems Inc.); the nucleotide sequences of the primers used are described in Table S1. Relative expression values of the different genes were calculated from the threshold cycle (Ct) following the 2^–ΔΔCt^ method; *GAPDH* was chosen as a reference gene, after previous RefFinder algorithm analysis using *GAPDH*, *B2M* e *RNA18S*.

### 2.5. Protein Extraction and Western Blotting for Protein Expression Analysis

SH-SY5Y cells were processed to obtain a cytosolic protein fraction (for synapsin 1 and tyrosine hydroxylase) or a total membrane protein fraction (for GLUT4) as previously described [50]. Moreover, for CREB/ICER analysis, the cells were homogenized, submitted to 1000× *g* centrifugation, and the supernatant was used as a total cellular protein fraction. The total protein content quantification was performed by Bradford method according to manufacturer recommendations (Bio-Rad Laboratories, Hercules, CA, USA) and equal amounts of protein (10 µg) were electrophoresed, transferred into the nitrocellulose membrane, and immunoblotted. The details of the primary antibodies used are presented in Table S1. The appropriate secondary conjugated antibody was used according to manufacturer specifications, followed by enhanced chemiluminescence (ECL) procedure using SuperSignal^®^ West Pico Plus Chemiluminescent Substrate (ThermoScientific, Rockford, IL, USA). The images were taken using Syngene automated system model G:BOX Chemi XRQ (Synoptics, Cambridge, UK) and the optical density of the blots was analyzed using Image J software (National Institutes of Health, Bethesda, MD, USA version 1.51). The nitrocellulose membrane was stained by Ponceau and the densities of the respective lanes were used for normalization. The results were expressed as arbitrary units, related to mean of the controls, which was set as 1.0.

### 2.6. Chromatin Immunoprecipitation Assay (ChIP Assay) for p65 Analysis

The total chromatin from SH-SY5Y cell was obtained using High-Sensitivity ChIP Kit (Abcam, UK, #ab185913) and all procedures were performed according to manufacturer recommendations, except the chromatin shearing step, which was empirically optimized and performed by ultrasound sonication procedure, consisting of 10 µg of total chromatin, diluted in 500 µL of lysis buffer, subjected to 15 pulses of 30 s ON/OFF at amplitude 35% using Vibra-Cel^®^ Ultrasonic Liquid Processors model VCX-130 (Sonics & Materials, Inc., Newtown, CT, USA). The electrophoresed chromatin in agarose gel (1%) was stained with UniSafe Dye according to manufacturer recommendations (Uniscience Corp., Sao Paulo, Brazil) and visualized under ultraviolet light using Syngene automated system model G:BOX Chemi XRQ (Synoptics). The length of sheared DNA obtained was between 100 and 700 bp with a peak size of 300 bp. The purified DNA obtained was analyzed by qPCR (Platinum^®^ SYBR^®^ Green qPCR SuperMix UDG, Invitrogen Life Technologies) using a StepOne Plus Instrument (Applied Biosystems Inc.). The antibodies and nucleotide sequence primers used are described in detail in Table S1. The results were calculated by the fold-enrichment (FE) method using the following formula: FE% = 2 (IgG CT-Sample CT) × 100%.

### 2.7. Statistical Analysis

Data were expressed as mean ± standard error of the mean (SEM); continuous variables were expressed as mean ± standard deviation (SD). Comparison between two groups was performed using unpaired Student’s *t*-test. Comparisons among 3 or 4 groups were performed by one-way analysis of variance (ANOVA), followed by Tukey test, after confirming the normality of the data distribution by Shapiro–Wilk test. Correlations between two variables were analyzed by Pearson (r) or Spearman (ρ) correlation coefficient analysis, according to the normal distribution of the data. In the time-course analysis, data were initially analyzed by two-way analysis of variance (ANOVA) to verify the effect of time, treatment, and interaction; afterward, the effect of the treatment was analyzed in each time-point by the unpaired Student’s *t*-test. Comparisons were considered statistically significant at *p* < 0.05. Analyses were performed using GraphPad Prism 9. Determination of the sample size can be seen in detail in the Supplementary Materials.

## 3. Results

### 3.1. Clinical and Demographic Characteristics of the Subjects Studied

Based on their BMI and history of DM, forty-one subjects were categorized into three groups: control (C), obese (OB), and obese with DM (OBDM), as shown in Table 1. BMI was the only continuous variable significantly different, pointing out that the mean value of the control group indicated the presence of overweight, and that the obesity grade was similar in obese groups with or without DM. As to DM subjects, considering their advanced age and obesity, we can highly suppose that they had type 2 DM; although, this cannot be asserted based on medical diagnosis.

### 3.2. GLUT4 Protein Detection in Hippocampus of Human Brain

**Figure 1:**
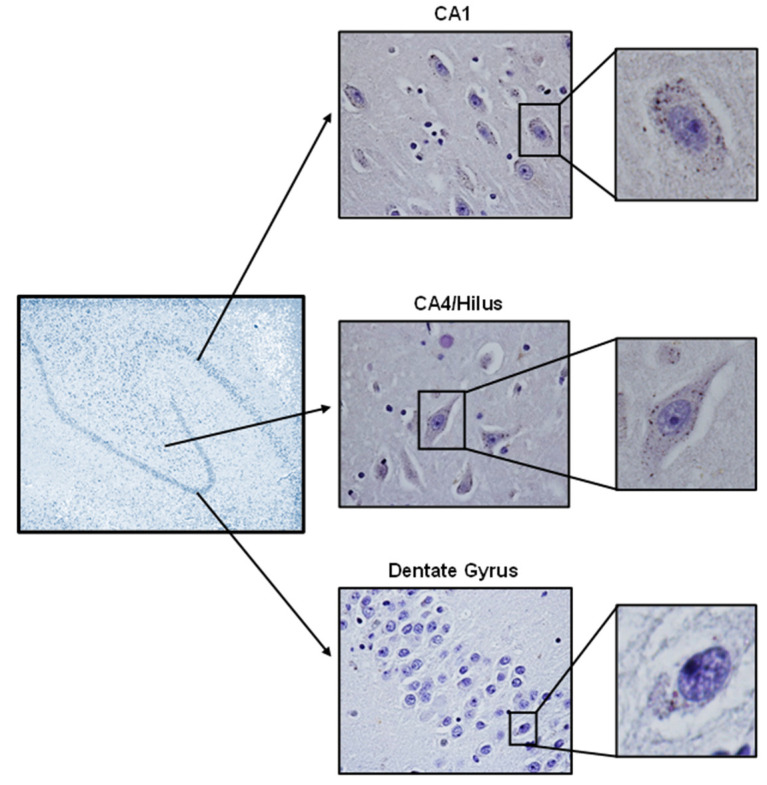
GLUT4 protein detection in hippocampus of human brain. GLUT4 was immunodetected in hippocampal neurons from the cornu ammonis (CA) CA1, CA4 (hilus), and dentate gyrus areas. As indicated in the figure, each hippocampal area was amplified, and from each area, a representative GLUT4-stained neuron was shown. High neuronal GLUT4 immunoreactivity was observed in CA1 and CA4 areas, and GLUT4 immunoreactivity was observed mainly in the intracellular neuronal body. Images were captured with the 4x, 40x, and 400x objectives, respectively, from left to right.

Immunodetection of GLUT4 in the hippocampus of a control subject (non-obese and non-DM) can be seen in Figure 1. The images reveal a higher content of GLUT4 in the cornu ammonis (CA) areas CA1 and CA4/hilus, mainly in the intracellular neuronal body. Considering the large (and amenable to study) cellularity of CA4/hilus, this area was chosen for further analysis.

### 3.3. Diabetes Decreases Neuronal GLUT4 Protein Content and Soma Area in Hippocampal CA4/Hilus Area

**Figure 2:**
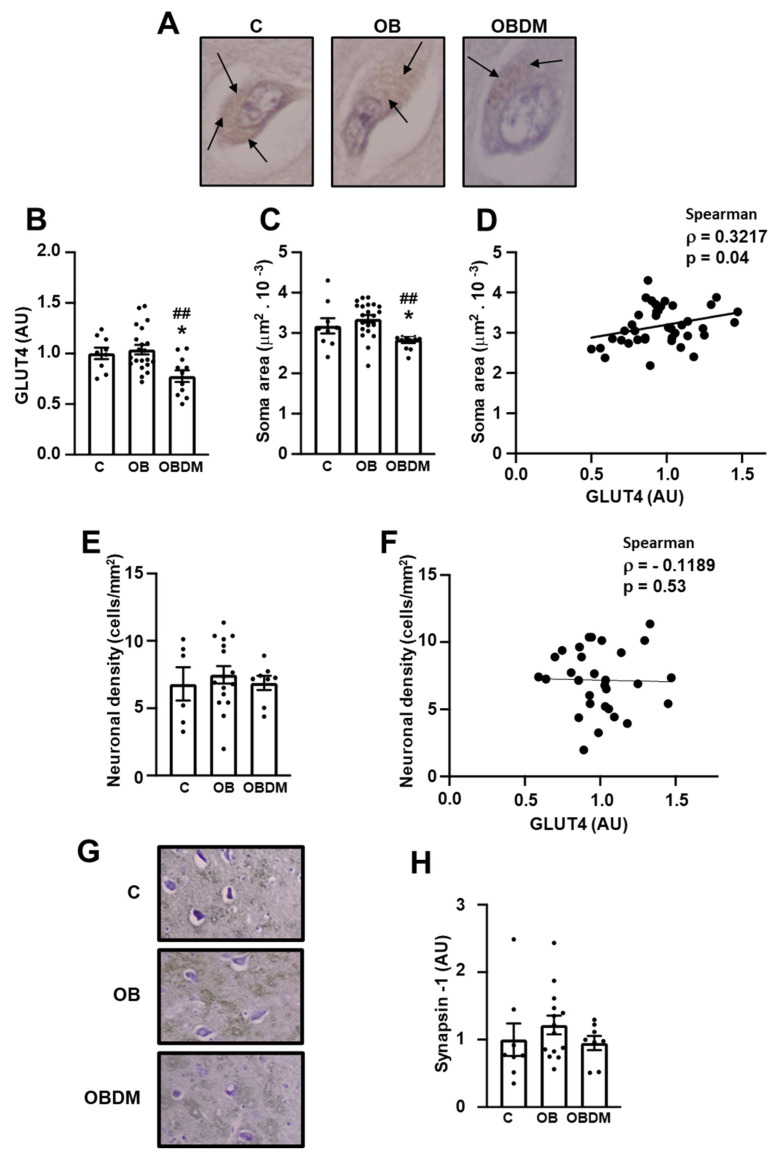
Diabetes decreases neuronal GLUT4 protein content and soma area in the hippocampal CA4/hilus area. Neuronal GLUT4 content and potential neuronal degenerative signals were analyzed in hippocampal CA4/hilus area from overweight (**C**), obese (OB), and obese diabetic (OBDM) subjects: (**A**) representative images of GLUT4 stained neurons (arrows indicate immunostained GLUT4); (**B**) GLUT4 quantification; (**C**) neuronal soma area; (**D**) correlation between neuronal soma area and GLUT4 content; (**E**) neuronal density; (**F**) correlation between neuronal density and GLUT4 content; (**G**) representative images of synapsin-1; (**H)** synapsin-1 quantification. Data are expressed as mean ± SEM of 9 to 21 (**B**,**C**,**E**) and 8 to 13 (**H**) subjects, and were analyzed by one-way ANOVA followed by Tukey’s multiple comparisons test; * *p* < 0.05 vs. C, ## *p* < 0.01 vs. OB. In (**D**), Spearman correlation test was used. Images were captured with the 400x objective.

Figure 2A shows representative images of GLUT4-stained neurons from C, OB, and OBDM subjects. Neuronal GLUT4 content (Figure 2B) was reduced by ~25% in OBDM subjects (*p* < 0.05 vs. C, *p* < 0.01 vs. OB); moreover, neuronal soma area (Figure 2C) was also reduced in the hippocampus from OBDM subjects (*p* < 0.05 vs. C and *p* < 0.01 vs. OB). Importantly, analysis of data from all three groups of subjects shows a positive correlation (*p* = 0.04) between GLUT4 content and neuronal soma area (Figure 2D). Neuronal density (Figure 2E) and its correlation with GLUT4 content (Figure 2F) were both unaltered, and so was SYN1 protein expression (Figure 2G,H).

### 3.4. Obesity Increases NFKB Subunit p65 but Does Not Change Carboxymethyllysine (CML) Content in Neurons from Hippocampal CA4/Hilus Area

**Figure 3:**
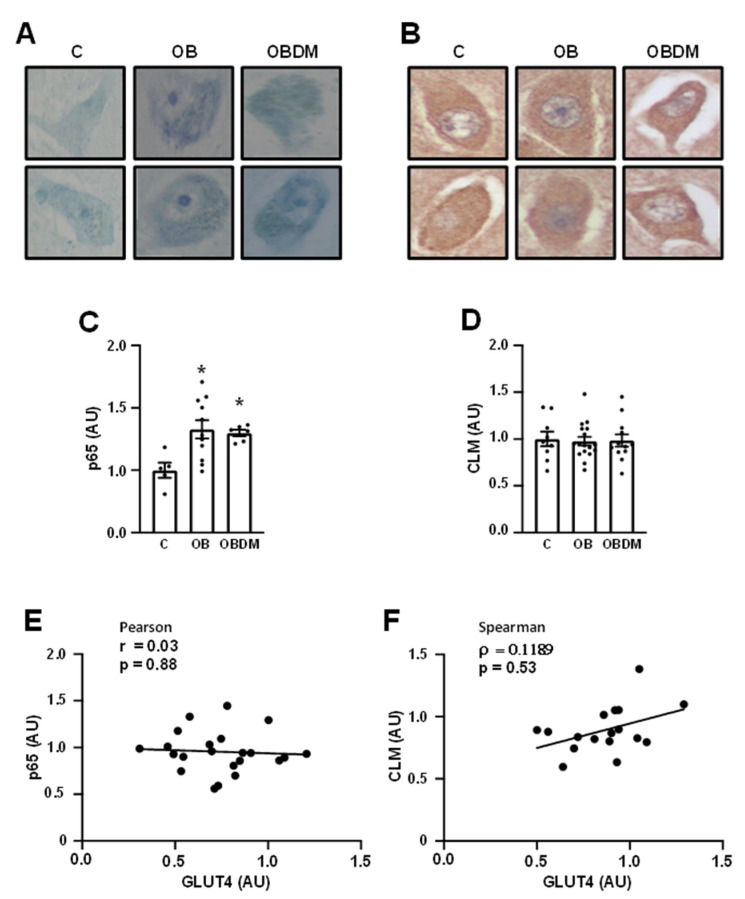
Obesity increases NFKB subunit p65 but does not change the carboxymethyllysine (CML) content in neurons from hippocampal CA4/hilus area. NF-kappa-B subunit (p65) and CML content were analyzed in hippocampal CA4/hilus area from overweight (**C**), obese (OB), and obese diabetic (OBDM) subjects. (**A**,**B**) illustrative images of p65 and CML stained neurons, respectively; (**C**) p65 quantification; (**D**) CML quantification; (**E**) correlation between GLUT4 and p65 contents; (**F**) correlation between GLUT4 and CML contents. Data are expressed as mean ± SEM of 6 to 11 (**C**) and 9 to 15 (**D**) subjects, and were analyzed by one-way ANOVA followed by Tukey’s multiple comparisons test; * *p* < 0.05 vs. C. In (**E**,**F**) individual data are shown, and Pearson and Spearman correlations (24 and 18 pairs, respectively) are shown. Images were captured with the 400x objective.

To investigate possible mechanisms involved in the repression of GLUT4 and reduction in the neuronal soma area in the brain from OBDM subjects, two molecular markers of inflammation and AGEs generation were selected for analysis: p65 and CML. Immunodetection of p65 (Figure 3A) revealed increased immunoreactivity in neurons from both OB and OBDM subjects, and quantification analysis (Figure 3C) confirmed the significant increase (*p* < 0.05) in p65 in both OB and OBDM groups (*p* < 0.05 vs. C); however, this marker showed no correlation with the GLUT4 content (Figure 3E). On the other hand, CML analysis (Figure 3B,D) revealed no change among the groups and no correlation with GLUT4 content (Figure 3F).

### 3.5. TNF Represses SLC2A4/GLUT4 Expression by a NFKB-Mediated Mechanism in Human Neuronal SH-SY5Y Cells

**Figure 4:**
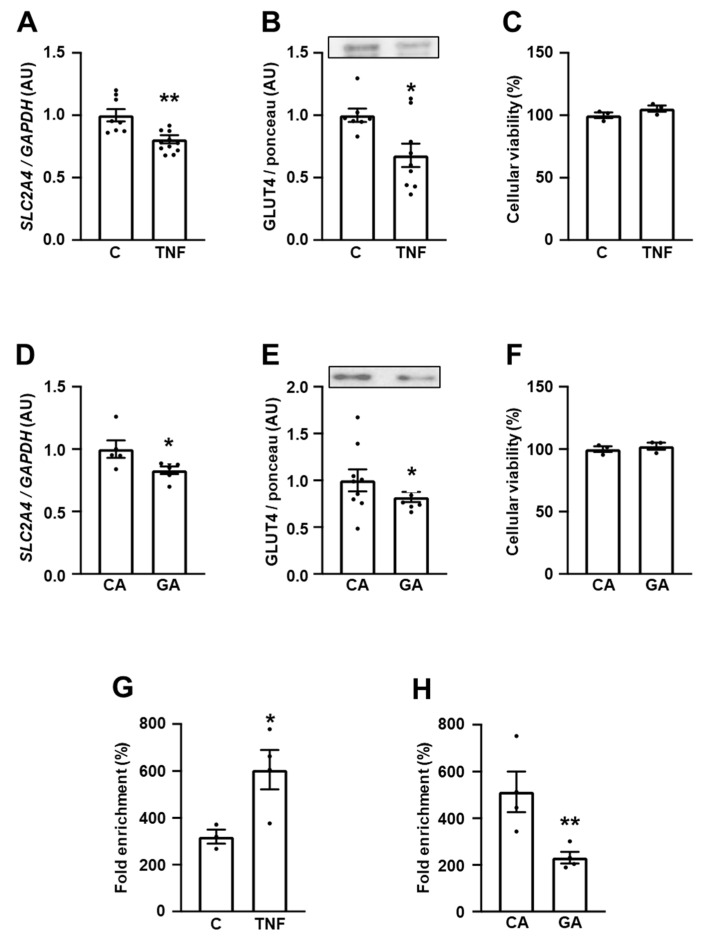
TNF represses *SLC2A4*/GLUT4 expression by an NFKB-mediated mechanism in human neuronal SH-SY5Y cells. *SLC2A4*/GLUT4 expression was evaluated in human neuronal SH-SY5Y cells treated with 20 ng/mL TNF for 3 h (**A**–**C**,**G**) or 0.4 mg/mL GA for 24 h (**D**–**F**,**H**). (**A**,**D**) *SLC2A4* mRNA normalized by *GAPDH* mRNA; (**B**,**E**) GLUT4 protein normalized by the optical density of the respective Ponceau stained lane, representative GLUT4 blots are shown at the top; (**C**,**F**) cell viability expressed as % of control; (**G**,**H**) NFKB (p65) binding activity in the *SLC2A4* promoter region expressed as % of fold enrichment. Data are expressed as mean ± SEM of 6 to 9 (**A**,**B**,**D**,**E**) or 5 (**C**,**F**–**H**) samples, and were analyzed by unpaired Student’s *t*-test; * *p* < 0.05 and ** *p* < 0.01 vs. C or CA. In the figures: C, control; TNF, tumor necrosis factor; CA, control albumin; GA, glycated albumin.

For a more thorough investigation of the possible participation of inflammation and AGEs generation upon repression of *SLC2A4*/GLUT4 expression in neurons, we investigated the effects of TNF and GA in SH-SY5Y cells. TNF significantly reduced the *SLC2A4* mRNA (Figure 4A) by ~20% (*p* < 0.01) and GLUT4 (Figure 4B) by ~32% (*p* < 0.05), and GA reduced the *SLC2A4* mRNA (Figure 4D) and GLUT4 (Figure 4E) by ~20% (*p* < 0.05); both treatments did not cause alterations in cellular viability (Figure 4C,F).

To confirm the involvement of inflammation in those regulations and considering the activation of NFKB, a proinflammatory effector already known as a mediator of *Slc2a4* gene repression induced by both TNF [47] and GA [50], we evaluated the NFKB (p65) binding activity in the *SLC2A4* promoter region. The p65 binding activity into the *SLC2A4* promoter in response to 3-h TNF incubation (Figure 4G) almost doubled (*p* < 0.05), revealing its participation in the repression of *SLC2A4*; however, surprisingly, 24-h GA treatment (Figure 4H) decreased the p65 binding activity by 40% (*p* < 0.01).

### 3.6. TNF Represses the Expression of Neuronal Markers in Human Neuronal SH-SY5Y Cells

**Figure 5:**
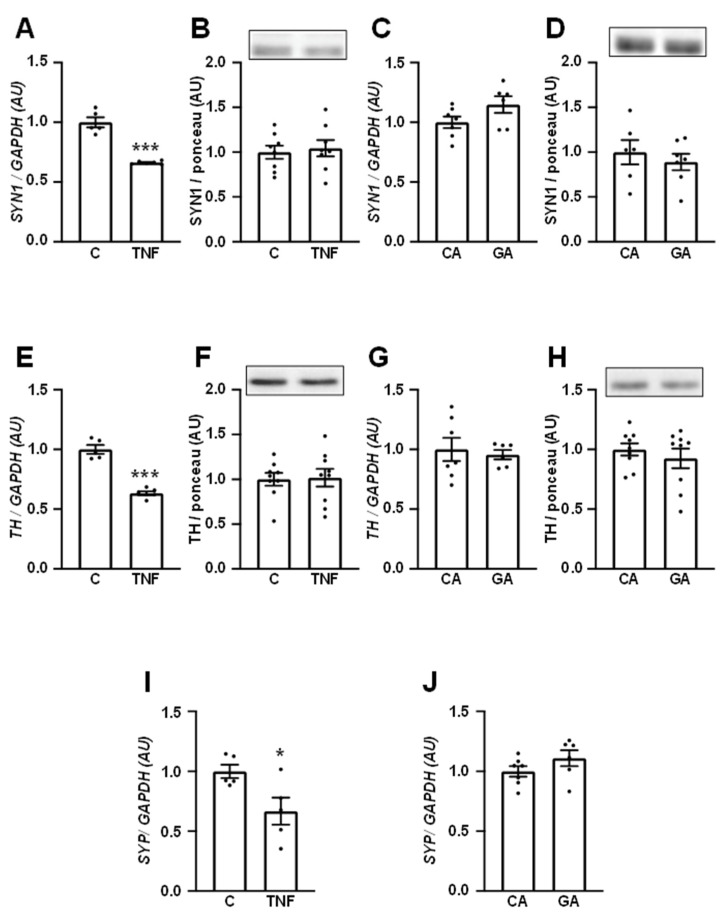
TNF represses the expression of neuronal markers in human neuronal SH-SY5Y cells.The expression of synapsin-1 (**A**,**C**), tyrosine hydroxylase (**E**,**G**) and synaptophysin (**I**,**J**) mRNAs, and the expression of synapsin-1 (**B**,**D**) and tyrosine hydroxylase (**F**,**H**) proteins were evaluated in human neuronal SH-SY5Y cells treated with 20 ng/mL TNF for 3 h or 0.4 mg/mL GA for 24 h. Data are expressed as mean ± SEM of 5 to 9 samples, and were analyzed by unpaired Student’s *t*-test; * *p* < 0.05 and *** *p* < 0.001 vs. C or CA. *SYN1,* synapsin-1 mRNA (SYN1 protein); *TH*, tyrosine hydroxylase mRNA (TH protein); *SYP*, synaptophysin mRNA; C, control; TNF, tumor necrosis factor; CA, control albumin; GA, glycated albumin.

SYN1 (Figure 5A–D) and TH (Figure 5E–H) mRNAs and protein expression, as well as *SYP* mRNA, were evaluated in SH-SY5Y cells treated with TNF (Figure 5A,B,E,F,I) for 3 h or with GA (Figure 5C,D,G,H,J) for 24 h. TNF clearly decreased the expression of *SYN1*, *TH,* and *SYP* mRNAs; however, the content of the respective proteins was unaltered, probably due to the short duration (3 h) of TNF treatment. Twenty-four-hour treatment with GA did not alter the investigated markers. It was not possible to detect SYP protein by Western blotting.

### 3.7. Inhibition of Histone Deacetylase 3 (HDAC3) Increases SLC2A4 Expression, Abrogates TNF-Induced Re-Pression of SLC2A4 Repression and Enhances Nuclear Content of CREB in Human Neuronal SH-SY5Y Cells

**Figure 6:**
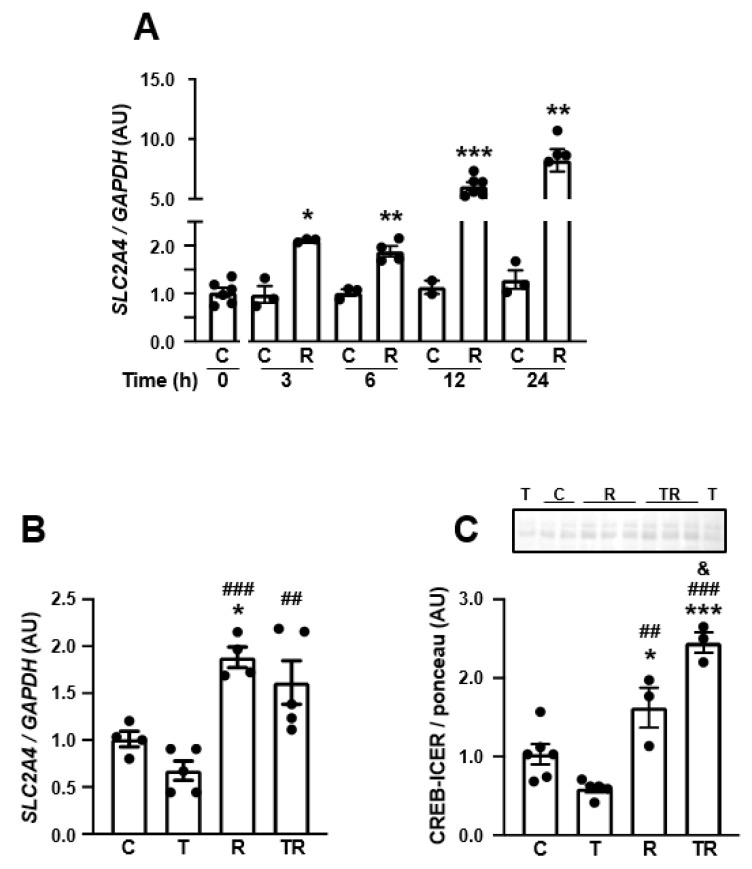
Inhibition of histone deacetylase 3 (HDAC3) increases *SLC2A4* expression, abrogates TNF-induced repression of *SLC2A4* repression, and enhances total cellular content of CREB in human neuronal SH-SY5Y cells: *(***A**) Time course of *SLC2A4* mRNA expression in human neuronal SH-SY5Y cells treated with 10 μM RGFP-966 (histone deacetylase 3 inhibitor) for 3 to 24 h. (**B**) *SLC2A4* mRNA expression and (**C**) cellular CREB-ICER content in response to 6 h of 20 ng/mL TNF and/or 10 μM RGFP-966 treatment. In (**A**) data are expressed as mean ± SEM of 4 to 6 samples, and were analyzed: in (**A**) by two-way ANOVA (time *p* < 0.0001, treatment *p* < 0.0001, interaction *p* < 0.001), followed by Student’s t-test between C and R in each time-point (* *p* < 0.05, ** *p* < 0.01 and *** *p* < 0.001 vs. C); in (**B**,**C**) data are expressed as mean ± SEM of 5 to 6 samples, and were analyzed by one-way ANOVA followed by Tukey’s multiple comparisons test (* *p* < 0.05, *** *p* < 0.001 vs. C, ## *p* < 0.01, ### *p* < 0.001 vs. T; & *p* < 0.05 vs. R). (**C**) control; R, RGFP-966; T, tumor necrosis factor; CREB, cAMP response element-binding protein; ICER, inducible cAMP early repressor.

DM has been reported to induce several epigenetic regulatory processes. Among these regulations, lysine acetylation of histone 3 (H3Kac) is one of the most common and has already been reported to be triggered by DM in the *Slc2a4* gene. Thus, we investigated the effect of increasing lysine acetylation of histones (HKac) by treating the SH-SY5Y neurons with the HDAC3 inhibitor RGFP-966. The results showed that RGFP-966 increases *SLC2A4* immediately after 3 h (by twofold) of treatment, and this effect continues to increase up to 24 h (by eightfold) (Figure 6A). Additionally, we checked whether RFPG-966 might abrogate the repressor effect of TNF (Figure 6B), and the results have shown that the HDAC3 inhibition in the presence of TNF increased the *SLC2A4* expression by 150%, reaching a value ~2-fold higher than the control value after 6 h of treatment.

Finally, to verify whether HKac epigenetic regulation might have an effect on neurodegeneration, we investigated the effect of RFPG-966 in the neuronal content of CREB/ICER (CRE-binding proteins), which are known to play a fundamental role in neuronal survival and plasticity. The results have shown (Figure 6C) that HDAC3 inhibition increased the neuronal content of CREB/ICER by 245% after 6 h of culture; in addition, the reduction in CREB/ICER induced by TNF was completely overbalanced by RFPG-966, reaching a value even higher than that observed under control conditions (*p* < 0.01 vs. TNF; *p* < 0.05 vs. C).

## 4. Discussion

Diabetes mellitus (DM) has emerged as a risk factor for the development of memory loss and brain damage; however, the mechanisms involved are still unclear. Metabolic disturbances, impairing neuronal glucose supply in the hippocampus, have been highlighted as important to the development of memory [19]; in this respect, the insulin-sensitive glucose transporter GLUT4, which, in the brain, expresses mainly in hippocampal neurons, has been shown to play a key role in the hippocampal memory [56]. Thus, the regulation of GLUT4 expression in the hippocampal neurons of DM subjects is the focus of this study.

To the best of the authors’ knowledge, this is the first study in which a GLUT4 analysis in postmortem human hippocampal tissues has been performed. GLUT4 immunoreactivity was strongly detected in the CA1 and CA4/hilus areas and, by considering the easier access for cell counting and analyzing in CA4/hilus, this area was selected for GLUT4 quantification and further analysis. GLUT4 immunoreactivity was reduced in CA4/hilus from OBDM subjects, something not observed in non-DM obese subjects, suggesting that some DM-related metabolic/hormonal conditions might be triggering that regulation. Importantly, the neuronal soma area was also reduced in DM subjects, and a positive correlation between GLUT4 content and soma area was observed, indicating that the impairment of glucose supply may be compromising neuronal homeostasis and imposing some cellular damage, as has been observed in several other conditions of chronic brain energy failure [30].

It is well known that GLUT4 expression decreases in muscle and adipose tissues in insulin-resistant conditions such as DM [55]; in this process, some potent repressor mechanisms of the *SLC2A4* gene expression have been raised, such as the inflammatory cytokine TNF, via the NFKB pathway, and the AGEs, via endoplasmic reticulum stress/inflammation [58]. Thus, we evaluated the expression of two markers of these effectors in the CA4 area, the p65 subunit of the NFKB and carboxymethyllysine (CML). NFKB (p65) was similarly increased in both OB and OBDM subjects, indicating a pro-inflammatory activity preceding the development of DM. Despite the powerful repressor effect of NFKB upon *SLC2A4* gene expression [47] and its clear involvement in the reduction in GLUT4 in DM [48,50,51], the p65 immunoreactivity did not correlate with the GLUT4 expression, indicating the involvement of other genetic or even epigenetic mechanisms. Moreover, no alteration in the CML expression was observed, suggesting that, at least in the hippocampus, the advanced glycation was not strongly activated in the DM subjects studied.

For a more thorough investigation of the DM-induced repressive mechanisms of the *SLC2A4* gene, human neuronal cells SH-SY5Y were challenged with TNF and glycated albumin (GA). The TNF and GA concentrations and time of exposure were in the range described as capable of reducing the *Slc2a4*/GLUT4 expression in adipocytes [59,60] and skeletal muscle slices [48,50]. Moreover, the TNF and GA concentrations and exposure times utilized were previously tested in rat neuronal PC-12 cells, when cellular viability was confirmed to be preserved (Figure S2). An SH-SY5Y-derived neuron was chosen because: (1) it has been extensively used for studies related to AD; (2) it expresses GLUT4 as the hippocampal neuron does, and (3) insulin-induced repression of GLUT4 expression and translocation has already been reported in SH-SY5Y cells [61]. 

In human SH-SY5Y cells, both TNF and GA were capable of repressing the *SLC2A4* mRNA and GLUT4 protein expression; an effect that was also observed in rat-PC-12-derived neurons (Figure S2). Furthermore, the participation of NFKB as a mediator of the TNF effect was confirmed by the increased binding activity of p65 in the *SLC2A4* promoter in the ChIP assay. The TNF-induced and p65-mediated repressor effect of *Slc2a4*/GLUT4 has already been described in adipocyte and skeletal muscle by an electrophoretic mobility assay [48,60]; however, this effect has never been reported in cultured human neurons.

The GA-induced decrease in the *SLC2A4*/GLUT4 expression was not accompanied by the increased binding activity of NFKB into the gene; on the contrary, the p65 binding activity was reduced in response to GA. Differently, 2.5-h incubation of skeletal muscle slices was reported to activate the canonical NFKB pathway, an effect that culminated in the increased nuclear content of NFKB p50 and p65 proteins, and in increased binding activity into the *Slc2a4* promoter [50]. It is important to notice that, in the in vitro analyses, we are looking for GA-induced inflammatory activation directly into the neuron, excluding effects that could be initiated in other cells such as the microglia, highlighting that, in the periphery, strong effects of AGEs are triggered in the macrophages, including the secretion of cytokines [58].

To investigate whether the DM-induced effectors TNF and AGE might be participating in neuronal dysfunction, as suggested by the reduction in the soma area of hippocampal neurons from DM subjects, some markers of neuronal function were evaluated in SH-SY5Y cells challenged with TNF or GA. Three-hour incubation of the neurons with TNF was capable of decreasing *SYN1*, *TH,* and *SYP* mRNAs; however, this duration of treatment was not enough to alter the SYN1 and TH protein content, pointing out that the repression of gene expression currently requires longer periods of time to reflect in the protein content, since the decrease in protein levels depends on their half-life (T1/2). For instance, synaptic-related proteins are known to have a long half-life, 4.95 and 4.09 days for SYN1 and SYP, respectively [62].

Differently, after the 24-h incubation of SH-SY5Y neurons with GA, despite the reduction in *SLC2A4*/GLUT4 expression, neither the mRNAs nor the proteins of the neuronal markers were altered. Neuronal degeneration and cell death were reported to occur in cortical neuronal cells, in response to 48-h incubation with GA at concentrations from 0.5 to 2.0 mg/mL [63]. Here, neurons were treated with 0.4 mg/mL GA for 24 h, and cell viability was completely preserved; thus, we can suppose that a longer exposure time and/or a higher concentration of GA would be necessary to start signals of neurotoxicity in SH-SY5Y cells.

DM and neurodegenerative diseases have been reported to induce several epigenetic regulations of gene expression, including histone post-translational modifications (PTMs). Among PTMs, lysine acetylation of histone 3 and 4 (H3Kac, H4Kac) are the most studied [64], and changes in H3Kac content have already been reported to be triggered by DM in the *Slc2a4* promoter, accompanying a reduction in muscle *Slc2a4*/GLUT4 expression [51,65]. Exposing neural stem cells to high glucose or hyperglycemia due to maternal DM in rats results in the decreased status of H3Kac and decreased transcriptional activity, leading to neural tube defects in the developing brain [66]; however, opposite regulation was reported for H4Kac status in neuroepithelial cells exposed to high glucose concentrations [67]. In this study, we investigated the effect of HDAC3 inhibition by treating SH-SY5Y cells with RGFP-966, expecting that it could modulate the expression not only of *SLC2A4*, but also of some neuronal markers.

In a time-course study, the incubation of neurons with RGFP-966 revealed a progressive increase in *SLC2A4* mRNA expression, which was eightfold higher at 24 h of treatment. This response indicates that the increased HKac, by facilitating the binding of preexisting enhancer transcription factors, can participate in the enhancement of the *SLC2A4* transcriptional activity in a direct way. However, with that experimental approach, the increased DNA HKac might also activate an indirect stimulatory effect upon the *SLC2A4* transcription. For instance, maternal protein restriction during pregnancy was reported to increase GLUT4 expression in female offspring skeletal muscle, which was related to both increased HKac at the *Slc2a4* promoter region (direct effect) and to increased expression of the *Slc2a4* enhancer myocyte enhancer factor 2A (indirect effect) [68].

Considering the TNF-induced and NFKB-mediated repressor effects described in SH-SY5Y neurons, we finally investigated whether the increased DNA HKac content could modulate the TNF effects upon both the *SLC2A4* gene and neuronal marker genes. A six-hour incubation of SH-SY5Y with RGFP-966 doubled *SLC2A4* mRNA expression and this effect was maintained despite the addition of TNF in the culture. These results reveal that the TNF-induced repression of *SLC2A4* transcription was completely counterbalanced by RGFP-966 treatment, and uncover HDAC3 inhibition as a promising target to improve GLUT4 expression in neurons. It is important to point out that, in this set of experiments (Figure 6B), the decreasing effect of TNF upon *SLC2A4* mRNA was not statistically significant (*p* = 0.0958), as it was in the previous set of experiments (Figure 4A, *p* < 0.01); however, the TNF repressor effect in Figure 6B (by ~35%) was even higher than that observed in Figure 4A (by ~20%). These apparently divergent results may be explained by the distinct statistical analysis performed: only two groups were compared by Student’s *t*-test (Figure 4A), whereas four groups were compared by one-way ANOVA, two of them with very high mean values, which is known to make the analysis more difficult to reach statistical significance (Figure 6B).

Regarding the investigation of a potential epigenetic regulation of the expression of neuronal markers of function and survival in cells treated with RGFP-966, a global analysis was chosen to measure the CRE-binding proteins CREB/ICER, which play an important role in the expression of several genes related to neurodegenerative diseases [69]. Indeed, in a study conducted to determine common upstream transcription factors governing molecular networks associated with altered AD-related genes (ADGs) in AD brains, the molecular network analysis identified CREB as a central transcription factor; and to confirm that, an immunohistochemical study was conducted, revealing the abnormal accumulation of phosphorylated CREB in granules of granulovacuolar degeneration (GVD) in hippocampal neurons of AD brains [69]. That led the authors to propose CREB-mediated gene regulation as a molecular biomarker of the AD-related pathological process [70].

The present results revealed that, by inhibiting HDAC3 (i.e., increasing histone acetylation status), a significant increase in the total neuronal CREB/ICER content was observed in neuronal cells. Curiously, RGFP-966 was capable not only of reversing the tendency of TNF to reduce CREB/ICER, but also of inducing an additional significant increase in CREB/ICER, indicating a complex interaction between NFKB activation and increased DNA HKac. Interestingly, the in silico analysis revealed that the second-rank pathway related to ADG regulation was the transcriptional regulation by NFKB [70]. Nevertheless, a limitation of our study is that it did not assess the nuclear content of CREB/ICER, to confirm the real CRE-activated transcriptional activity, pointing out that sequestration of CREB in granules of GVD in the hippocampal neurons of AD brains was described as being accompanied by a reduction in CREB nuclear content [70]. Moreover, interpreting the CREB-regulated transcriptional network is still considerably intricate, since it comprises 17 upregulated ADGs, and also 26 downregulated ADGs, indicating neither the overactivation nor the hypoactivation of the CRE-mediated transcriptional activity; revealing, this way, a widespread deregulation of this signaling pathway in the pathophysiology of AD [70]. It is worth emphasizing that regarding the two genes analyzed here, while the *SLC2A4* expression is enhanced [71], *SYN1* is repressed [70] by CREB/ICER, reinforcing that both up- and down-regulation of ADGs may be associated with the CREB/ICER deregulated activity.

Overall, this study reveals that the DM-induced reduction in the hippocampal GLUT4 content of humans is accompanied by increased proinflammatory activity; further, in isolated human neurons, inflammatory activity decreases the expression of *SLC2A4* and, also, of some genes related to neurodegeneration. Furthermore, these effects may be related to the epigenetic regulation (H3Kac and H4Kac) of the genes studied, since the *SLC2A4* mRNA and CREB/ICER expression can be enhanced by the inhibition of HDAC3. These data uncover the inhibition of deacetylase as being a promising target for the treatment of DM-related neurodegeneration.

Regarding HKac, the overexpression of beta-amyloid in an AD yeast model has been shown to decrease H3K9ac [72], and HDAC inhibition (increasing HK-ac status) was reported to improve the activation of transcription factor EB, promoting lysosome biogenesis, alleviating the pathogenesis of AD in a mouse model of AD [73]. This way, the hyperactivation of HDAC turns into a potential therapeutic approach for the treatment of memory loss and neurodegenerative diseases [74]. Moreover, an epigenome-wide study evaluating H3K9ac status in 669 aged human prefrontal cortices uncovers large-scale changes in H3K9ac driven by tau protein in aging and AD human brains; although, AD risk *loci* were not enriched in tau-associated H3K9ac domains; functional relevance of these chromatin changes was evinced by consistent transcriptional changes in three independent datasets, and similar findings in two mouse models of AD [75].

Both T1D and T2D display a proinflammatory status that converges into impaired glycemic control and long-term degenerative complications. In this respect, there is evidence of genetic association between DM and HDAC activity, and HDAC inhibitors have been reported to improve all these DM-associated conditions, and to positively affect late diabetic microvascular complications [75]. Because of this evidence, there is a strong rationale for preclinical studies and clinical trials aiming at testing the utility of HDAC inhibitors as a novel therapy for DM [76].

The scarcity of human hippocampus specimens (a postmortem tissue) led us to seek the material at the São Paulo Autopsy Service (a public service), which determined some special characteristics in the studied groups, such as the presence of obesity in DM subjects. That might be a limitation of this study, but it depicts the profile of the elderly DM subjects who have been autopsied in the City of São Paulo. Moreover, this is in accordance with that observed in T2DM cohorts from developed countries [77] and large cities [78], in which the prevalence of overweight/obesity is now 90%. Another limitation of our study was the absence of investigation regarding the possible participation of amyloid beta accumulation in this process; although, some studies have demonstrated that impaired hippocampal insulin signaling with reduced GLUT4 expression precedes amyloid-beta accumulation [79].

The most important result in this study is to reveal, for the first time, the association of DM/inflammation with the reduction in GLUT4 in the human hippocampus and isolated human neurons. In this regard, approaches to improve GLUT4 should be a promising target to preserve hippocampal neuronal homeostasis. In a recent review, based on several in vitro and in vivo studies, the capacity of metformin to improve GLUT4 expression has strengthened its beneficial effect on peripheral insulin resistance [80]. Whether that also holds true for the association between central insulin resistance and cognitive dysfunction in DM should be considered. Regarding that, the effects of metformin, alone or in combination with donepezil (an acetylcholinesterase inhibitor), were investigated in the brain/hippocampus of DM rats. Metformin alone, and with a better response once associated with donepezil, had a beneficial effect on oxidative stress, inflammation, endoplasmic reticulum stress, and cholinesterase activity in the brain/hippocampus of DM rats [80]. Importantly, the brain GLUT4 content, which was reduced by DM, has been recovered by metformin, with an even higher response with the association of the two drugs [81]; thus, the authors propose that the combination metformin/donepezil should be considered for managing DM-associated dementia [81]. Indeed, a recent systematic review and meta-analysis, supported by the available evidence, revealed that metformin therapy decreased the risk of cognitive decline in DM subjects [82].

## 5. Conclusions

This study reveals reduced hippocampal GLUT4 content and neuronal soma area in the brain of DM subjects, accompanied by increased proinflammatory activity; in isolated human neurons, the effect of inflammatory activation by TNF was confirmed to reduce not only *SLC2A4* gene expression but also the expression of some genes related to neuronal function. Furthermore, these effects may be related to epigenetic regulation (H3Kac and H4Kac status), since they can be counterbalanced by inhibiting HDAC3. These results uncover the improvement in GLUT4 expression and/or the inhibition of HDAC3 as promising therapeutic targets to fight DM-related neurodegeneration.

## Figures and Tables

**Table 1:** Clinical and demographic characteristics of the studied subjects.

Variables	Control *n* = 9	Obese *n* = 21	Obese with DM *n* = 11
Age (years), mean ± SD	60.22 ± 9.52	61.95 ± 12.55	69.73 ± 10.54
BMI (kg/m^2^), mean ± SD	27.21 ± 1.49	31.99 ± 1.38 *	32.29 ± 1.67 *
Male, *n* (%)	3 (33.3)	11 (52.4)	4 (36.4)
Ethnicity, *n* (%)			
White	4 (44.4)	17 (80.9)	7 (63.6)
Brown	3 (33.3)	1 (4.8)	2 (18.2)
Black	2 (22.2)	3 (14.3)	2 (18.2)
Socioeconomic class, *n* (%)			
High	0 (0)	7 (33.3)	2 (18.2)
Middle	3 (33.3)	6 (28.6)	5 (45,5)
Low	6 (66.7)	8 (38.1)	4 (36.3)
Education (years), mean ± SD	5.33 ± 2.95	6.9 ± 3.91	5.45 ± 4.9
Dyslipidemia, *n* (%)	0 (0)	3 (14.3)	1 (9)
Smoking, *n* (%)	4 (44.4)	5 (23.8)	5 (45.5)
Alcohol use disorder, *n* (%)	3 (33.3)	1 (4.8)	0 (0)
Coronary arterial disease, *n* (%)	1 (11.1)	4 (19.0)	2 (18.2)
Hypertension, *n* (%)	8 (89)	16 (76.0)	9 (81.8)
Heart failure, *n* (%)	0 (0)	5 (23.8)	3 (27.3)
Cerebrovascular disease, *n* (%)	1 (11.1)	1 (4.8)	4 (36.4)
Alzheimer’s disease, *n* (%)	0 (0)	0 (0)	1 (9)

Continuous variables are expressed by mean ± SD; and compared by one-way ANOVA with Tukey post-test; * *p* < 0.001 vs. control. BMI, body mass index; DM, diabetes mellitus; *n*, number of subjects; (%), percentage inside the group.

## Data Availability

The datasets used and/or analyzed during the current study are available from the corresponding author upon reasonable request.

## Supplementary Materials

The following supporting information can be downloaded at: https://www.mdpi.com/article/10.3390/cells12091250/s1, Supplementary Material and Methods: Statistical analysis—determination of sample size; Supplementary Material and Methods: Description of histological analyses; Table S1: Primers used for SYBR^®^ real-time PCR (q-PCR) and antibodies used for chromatin immunoprecipitation (ChIP) assay, Western blotting and immunohistochemistry; Figure S1: Human SH-SY5Y cells differentiation; Figure S2: GLUT4 expression in rat neuronal PC-12 cells; Supplementary Results: Original images of the Western blotting and respective Ponceau stained membranes.

## Abbreviations

Abbreviations (and names) of genes and proteins are in accordance with the HGNC and UniProt databases, respectively.

AD	Alzheimer’s disease
ADGs	AD-related genes
AGEs	advanced glycation end products
BBB	blood–brain barrier
BAS	Biobank for Aging Study
BMI	body mass index
CA1	cornu ammonis 1 area
CA4	cornu ammonis 4 area
CHIP	chromatin Immunoprecipitation
CML	carboxymethyllysine
CNS	central nervous system
CREB/ICER	CRE-binding proteins
DAB	3-3-diaminobenzidine tetrahydrochloride
DM	diabetes mellitus
GA	glycated albumin
GLUT1	glucose transporter type 1 protein
GLUT3	glucose transporter type 3 protein
GLUT4	glucose transporter type 4 protein
GVD	granulovacuolar degeneration
H3Kac	lysine acetylation of histone 3
H4Kac	lysine acetylation of histone 4
H3K9ac	lysine 9 acetylation of histone 3
HDAC3	histone deacetylase 3
IHC	immunohistochemistry
IR	insulin resistance
MTT	tetrazolium salt 3-(4,5-dimethylthiazol-2-yl)-2,5-diphenyltetrazolium bromide
OB	obese subject
OBDM	obese diabetic subject
p65	nuclear factor NF-kappa-B subunit p65
PB	phosphate buffer
qPCR	quantitative polymerase chain reaction
RAGE	advanced glycation end product receptor
RGFP-996	HDAC3 inhibitor
SYP	synaptophysin protein
*SYP*	synaptophysin gene (human)
SYN1	synapsin-1 (protein)
*SYN1*	synapsin-1 gene (human)
*Slc2a4*	solute carrier family 2 member 4 gene (mouse)
*SLC2A4*	solute carrier family 2 member 4 gene (human
SPAS	Sao Paulo Autopsy Service
TNF	tumor necrosis factor
T1D	type 1 diabetes
T2D	type 2 diabetes
TH	tyrosine hydroxylase protein
*TH*	tyrosine hydroxylase gene (human)
