# Supplementary material for: Postmortem Brains from Subjects with Diabetes Mellitus Display Reduced GLUT4 Expression and Soma Area in Hippocampal Neurons: Potential Involvement of Inflammation
Source: Cells. 2023 Apr 25;12(9):1250. doi: 10.3390/cells12091250 (PMC10177173; doi:10.3390/cells12091250)

Supplementary Material

Postmortem Brains from Subjects with Diabetes Mellitus Display Reduced GLUT4 Expression and Soma Area in Hippocampal Neurons: Potential Involvement of Inflammation

Caio Yogi Yonamine [1,2], Marisa Passarelli [3,4], Claudia Kimie Suemoto [5], Carlos Augusto Pasqualucci [6], Wilson Jacob-Filho [5], Venâncio Avancini Ferreira Alves [7], Suely Kazue Nagahashi Marie [8], Maria Lucia Correa-Giannella [9], Luiz Roberto Britto [1] and Ubiratan Fabres Machado [1,*]

1 Department of Physiology and Biophysics, Institute of Biomedical Sciences, University of São Paulo, São Paulo 05508-000, Brazil
2 Novo Nordisk Foundation Center for Basic Metabolic Research, Faculty of Health and Medical Sciences, University of Copenhagen, DK-2200 Copenhagen, Denmark
3 Laboratório de Lipides (LIM-10) do HCFMUSP, Faculdade de Medicina, Universidade de São Paulo, Sao Paulo 01246-000, Brazil
4 Programa de Pos-Graduação em Medicina, Universidade Nove de Julho (UNINOVE), São Paulo 01525-000, Brazil
5 Divisao de Geriatria, Faculdade de Medicina da Universidade de Sao Paulo, Sao Paulo 01246-000, Brazil
6 Departamento de Patologia, Universidade de Sao Paulo, Sao Paulo 01246-000, Brazil
7 Laboratório de Investigação Médica em Patologia Hepática, (LIM14) do Hospital das Clínicas (HCFMUSP) da Faculdade de Medicina, Universidade de São Paulo, São Paulo 01246-000, Brazil
8 Departamento de Neurologia, Faculdade de Medicina da Universidade de Sao Paulo, Sao Paulo 01246-000, Brazil
9 Laboratorio de Carboidratos e Radioimunoensaio (LIM-18) do Hospital das Clinicas HCFMUSP, Faculdade de Medicina, Universidade de Sao Paulo, Sao Paulo 01246-000, Brazil
* Correspondence: ubiratan@icb.usp.br

SUPPLEMENTARY MATERIAL AND METHODS

STATISTICAL ANALYSIS - DETERMINATION OF SAMPLE SIZE

As the GLUT4 analysis in hippocampus has never been conducted in humans, and there are only a few reports in experimental animals, it was difficult to find premises for calculating the sample size. The present calculation was based on the three groups we had (control, obese and obese+DM) and one study of GLUT4 expression in cerebellum of STZ diabetic rat (Vannucci et al., GLUT4 glucose transporter expression in rodent brain: effect of diabetes. Brain Res. 1998. 797:1-11). In the study by Vannucci et al, the GLUT4 expression was performed only in the cerebellum, which is also frequently affected by Alzheimer's disease. The calculation of sample size was performed as follows.

- The GLUT4 expression in diabetic rats was 1.7-fold lower than in non-diabetic rats;

- Considering the standard deviation observed in the study, we defined an alpha value of 0.05 and a beta error >0.8;

- With these premises, the number of samples (n) for each group should be at least 6, and the comparison of the means should be performed by One-Way ANOVA, followed by Tukey's multiple comparisons test, after confirming the normality of the data distribution by Shapiro-Wilk test.

For in vitro studies, we established a minimum of 5 duplicate samples, obtained from at least 4 different cultures. We reached this experimental number in all experiments, except for the time-course experiment of HDAC3 inhibition (Fig 6A), in which 3 control groups have only 4 samples; however, the statistical significance of the 2-way ANOVA was very high for all: time, treatment and interaction.

SUPPLEMENTARY MATERIAL AND METHODS

DESCRIPTION OF HISTOLOGICAL ANALYSES

GLUT4, CML and p65 analysis

Since the amount of material derived from humans was scarce, we have analyzed one slice per participant. On average, we have taken in average seven pictures per slice (participant). On average, each picture contained three to four neurons, totalizing approximately 28 neurons per participant per target. The images were analyzed with a 20X objective.

Below there is one picture containing four neurons. The yellow arrows indicate the GLUT4 staining. The red mark surrounding the neuron indicates the area for which we have measured the integrated density using the ImageJ software. We have subtracted the integrated density of area 2 from area 1, since area 2 was considered as the background. We have performed the same procedure for all other neurons. The same procedure was also used to analyze CML and p65.

The average of the integrated density of the stained area minus the integrated density of the background area was used for statistical analysis, and the protein content results were expressed as arbitrary units, related to mean of the controls, which was set as 1.0.

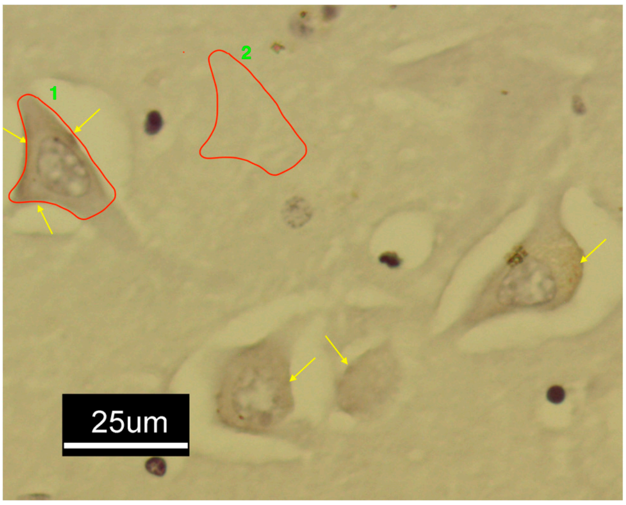

Synapsin-1 analysis

Since synapsin-1 is not detected inside the neuronal soma but instead in the axon terminals, we evaluated synapsin-1 immunostaining in a slightly different way in relation to the procedure described above. The images were analyzed with a 10X objective.

We have analyzed one slice per participant. We have taken 3 pictures per slice (participant). Each picture contained several synapsin-1 stained spots (30-40), totalizing over a hundred spots analyzed per participant per target. The total area analyzed was the same for all participants.

Below there is one image from a section stained for synapsin-1. The yellow arrows indicate the synapsin-1 staining. The red mark surrounding the stained region indicates the area from where we have measured the integrated density using the ImageJ software. We have subtracted the integrated density of area 2 from the integrated density of area 1, since area 2 was considered as the background. We have performed the same procedure for all other spots detected.

The sum of integrated density from the stained areas minus the background areas was used for statistical analysis, and the protein content results were expressed as arbitrary units, related to mean of the controls, which was set as 1.0.

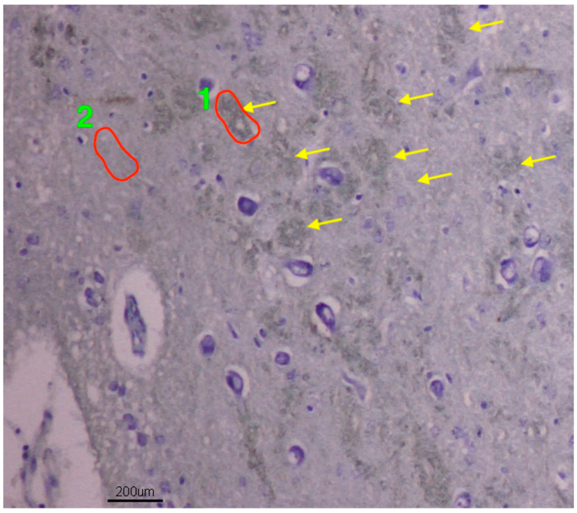

Neuronal size analysis

For the determination of neuronal size, we have used the function "area" using image J. As illustrated in the picture below, we have manually highlighted the neuron limits and measured the area. To obtain the precise value in µm^2, we have calibrated the ImageJ software according to the magnification ruler of the image acquisition system.

On average, we have analyzed 50 neurons per participant. Each dot shown in the graph from Figure 2C represents the average soma area (in µm^2) per participant (such as the one pointed by the red arrow).

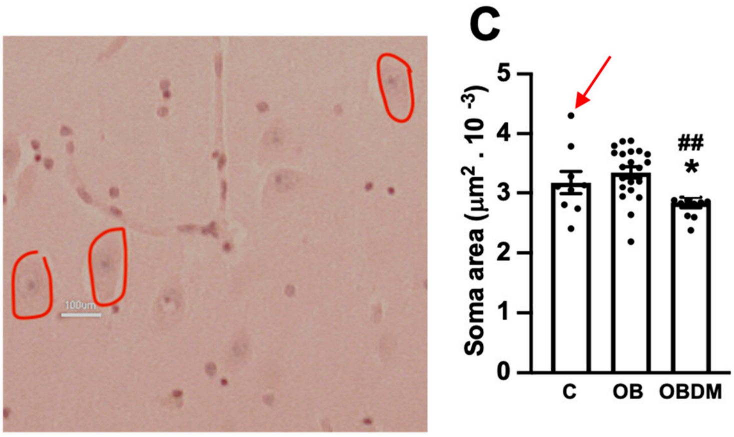

SUPPLEMENTARY Table S1. Primers used for SYBR® real-time PCR (q-PCR) and antibodies used for chromatin immunoprecipitation (ChIP) assay, Western blotting and immunohistochemistry.

Targets for qPCR	Primers (5`- 3`)	Sequences
SLC2A4 (human)	Forward	AGATTGCTCCCACTCACCTG
	Reverse	ATGATGTAGAGGTAGCGGGG
SYN1 (human)	Forward	TGACCAAGACGTATGCCACT
	Reverse	AATCTCTGAGCACGTGTCCA
SYP (human)	Forward	AATTCTTTGTCACCGTGGCC
	Reverse	TTCACATCTGACAGCCCCTT
TH (human)	Forward	AGATCGCCTTCCAGTACAGG
	Reverse	GATATTGTCTTCCCGGTAGC
GAPDH (human)	Forward	GGGTCTTTGCAGTCGTATGG
	Reverse	ACCTCCTGTTTCTGGGGACT
**Targets for ChIP-assay**		
SLC2A4 promoter region	Forward	GCCTTTTGTTCCAGGGACTC
(-185 to -53 segment)	Reverse	GAGTTTGGCTGGAGTTGGTG

Antibodies for ChIP-assay	Amount	Reference
RNA polimerase II	0.8 µg/reaction	Abcam 85913*
Non-immune IgG	0.8 µg/reaction	Abcam 85913*
p65	0.8 µg/reaction	Abcam ab7970
**Antibodies for Western blotting/IHC**		
GLUT4	1:3,000	Millipore #07-1404
CREB/ICER	1:1,000	Abcam 5803
SYN1	1:200	Millipore #AB1543
TH	1:200	Millipore #MAB318
p65	1:200	Abcam #ab7970
CML	1:2,000	Abcam #ab276684

IHC, immunohistochemistry; *SLC2A4*, solute carrier family 2 member 4; *SYN1*, synapsin 1; *SYP*, synaptophysin; *TH*, tyrosine hydroxylase; *GAPDH*, glyceraldehyde-3-phosphate dehydrogenase; p65, nuclear factor NF-kappa B subunity p65; GLUT4, glucose transporter 4; CREB, cAMP response element-binding protein; ICER, inducible cAMP early repressor; SYN1, synapsin 1; TH, tyrosine hydroxylase; CML, carboxymethyl lysine; * included in the high-sensitivity ChIP kit Abcam (ab185913).

SUPPLEMENTARY Figure 1

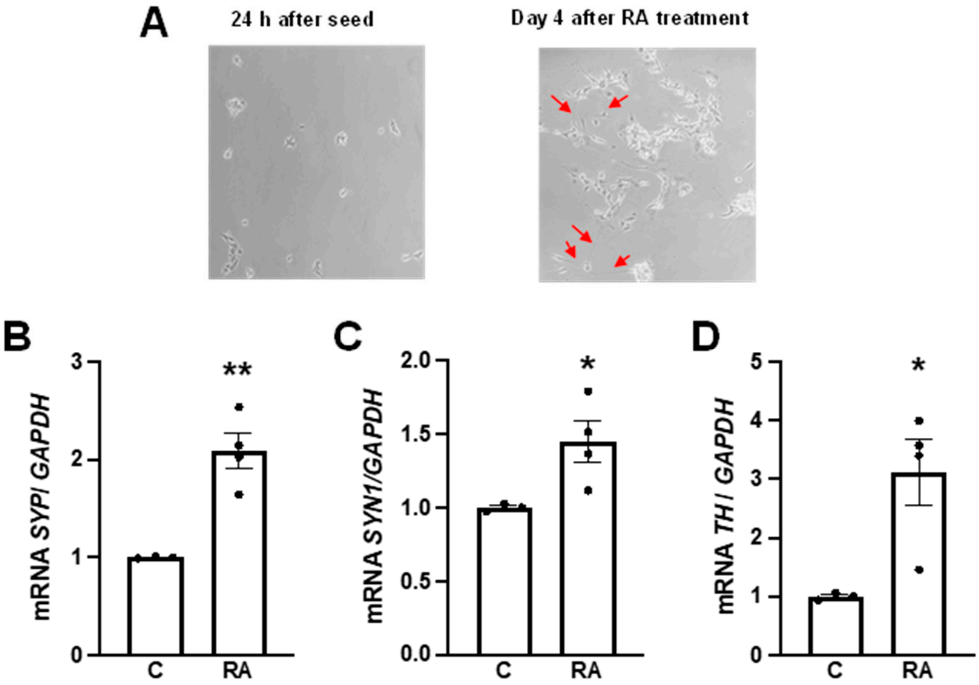

Figure S1. Human SH-SY5Y cells differentiation. Human SH-SY5Y cells were seeded and 24-hours later 10 µM retinoic acid was imposed (for 4 days) to induce neuronal differentiation. The efficiency of neuronal differentiation was confirmed by qPCR measurement of synaptophysin (*SYP*), synapsin-1 (*SYN1*) and tyrosine hydroxylase (*TH*) mRNAs, and by subjective identification of neurite outgrowth projections as shown in the upper panel. C, control condition (24-hour seeded cells); RA, 4-day retinoic acid treated cells (differentiated neurons).

SUPPLEMENTARY FIGURE 2

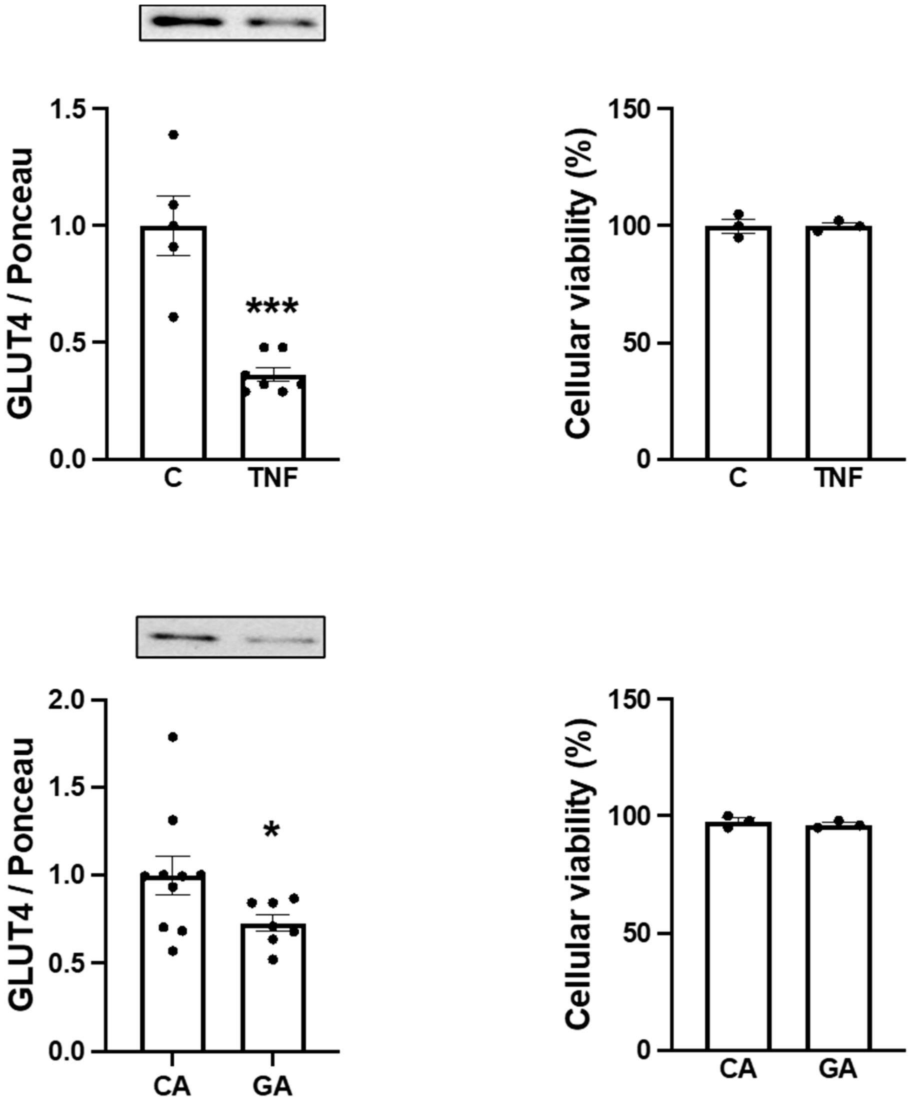
GLUT4 / Ponceau
1.5
1.0
0.5
0.0
***
C
TNF
Cellular viability (%)
150
100
50
0
C
TNF
GLUT4 / Ponceau
2.0
1.5
1.0
0.5
0.0
*
CA
GA
Cellular viability (%)
150
100
50
0
CA
GA

Figure S2 . GLUT4 expression in rat neuronal PC-12 cells. GLUT4 expression was analyzed in rat neuronal PC-12 cells treated with 20 ng/mL TNF for 3 hours (A and B) or with 0.4 mg/mL glycated albumin for 24 hours (C and D). GLUT4 protein was normalized by the optical density of the respective Ponceau stained lane. GLUT4 protein (A and C) and cell viability (B and D) data are expressed as mean ± SEM, and were analysed by Student t-test; *p < 0.05 and ***p < 0.001 vs. C or CA. In the figures: C, control; TNF, tumor necrosis factor; CA, control albumin; GA, glycated albumin.

SUPPLEMENTARY RESULTS

ORIGINAL IMAGES OF THE WESTERN BLOTTING AND RESPECTIVE PONCEAU STAINED MEMBRANES

Figure 4

GLUT4 in SH-SY5Y cells

Figure 4B

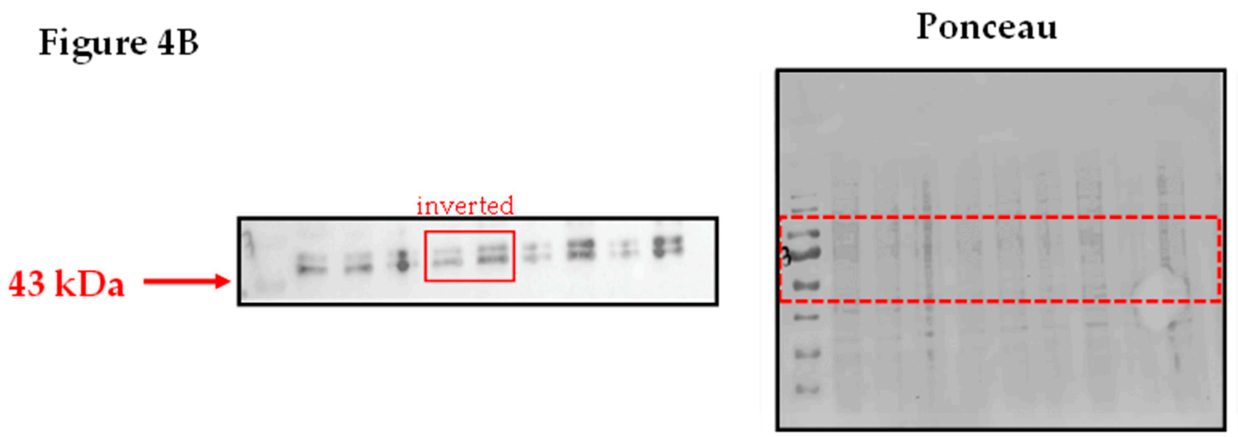

Figure 4E

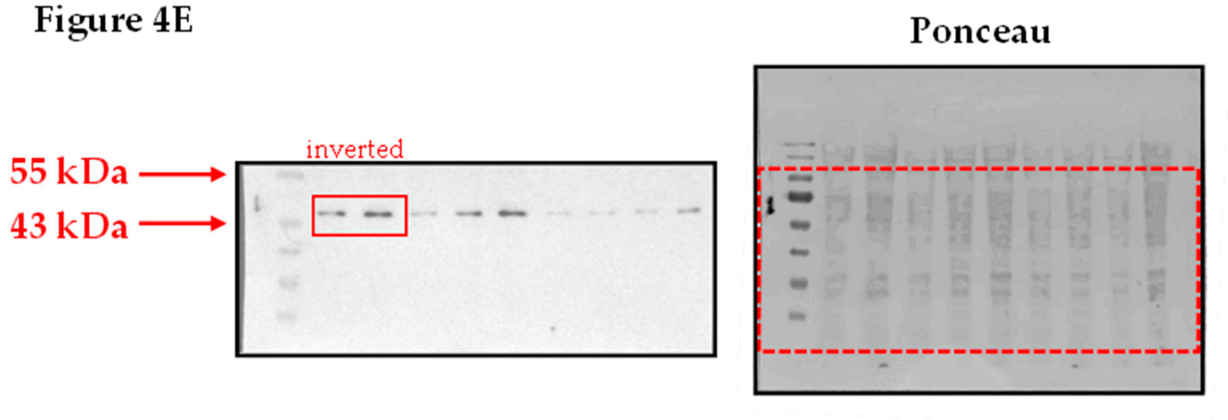

Figure 5

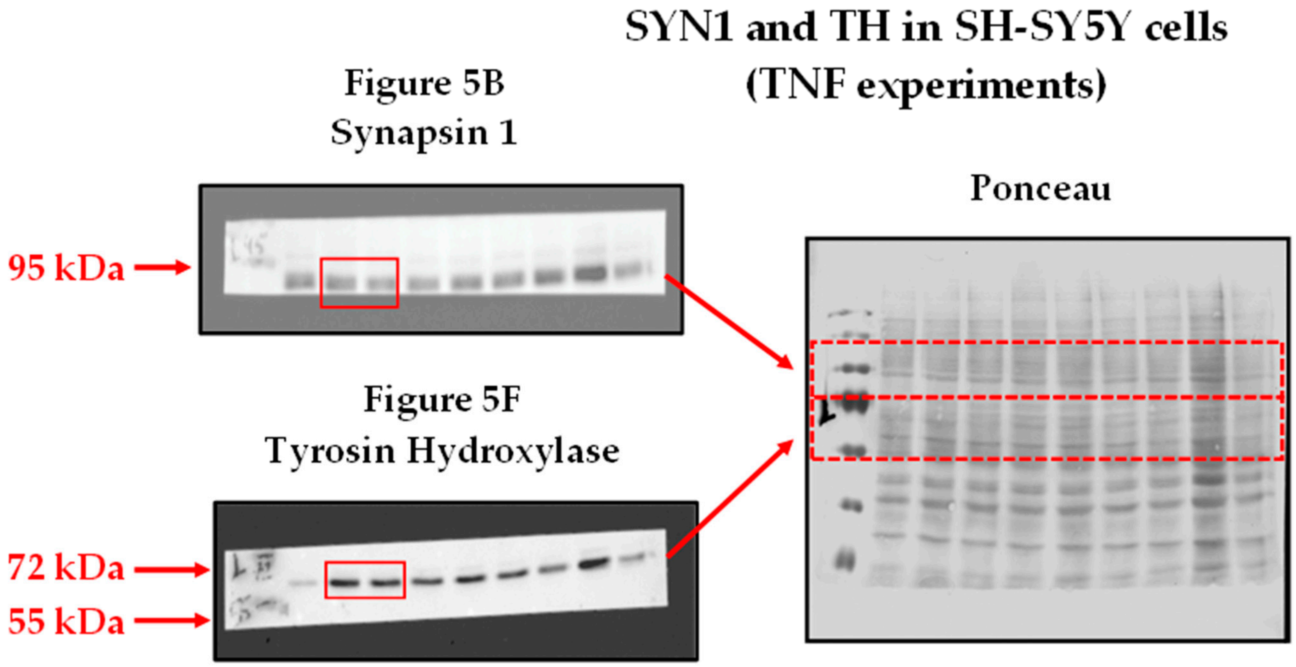
SYN1 and TH in SH-SY5Y cells
(TNF experiments)
Figure 5B
Synapsin 1
Ponceau
95 kDa
Figure 5F
Tyrosin Hydroxylase
72 kDa
55 kDa

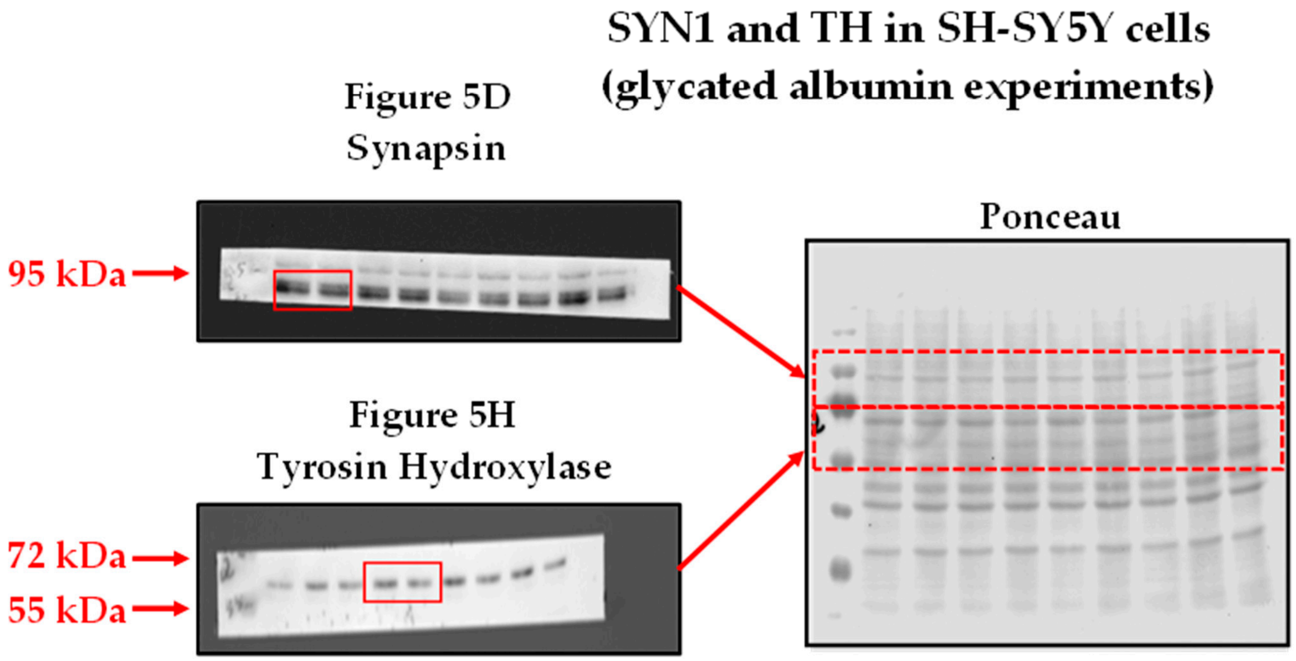
SYN1 and TH in SH-SY5Y cells
(glycated albumin experiments)
Figure 5D
Synapsin
Ponceau
95 kDa
Figure 5H
Tyrosin Hydroxylase
72 kDa
55 kDa

Figure 6C

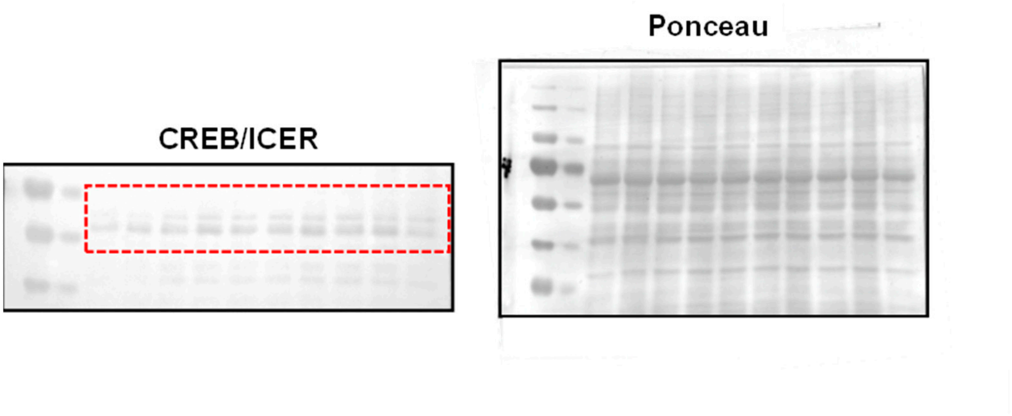

Supplement: Supplementary file 1 [file cells-12-01250-s001.zip › cells-2247262-supplementary.pdf]
